# Flexible Matrices for the Encapsulation of Plant Wearable Sensors: Influence of Geometric and Color Features on Photosynthesis and Transpiration †

**DOI:** 10.3390/s24051611

**Published:** 2024-03-01

**Authors:** Daniela Lo Presti, Sara Cimini, Francesca De Tommasi, Carlo Massaroni, Stefano Cinti, Laura De Gara, Emiliano Schena

**Affiliations:** 1 Unit of Measurements and Biomedical Instrumentation, Department of Engineering, Università Campus Bio-Medico di Roma, Vial Alvaro del Portillo 21, 00128 Roma, Italy; f.detommasi@unicampus.it (F.D.T.); c.massaroni@unicampus.it (C.M.); e.schena@unicampus.it (E.S.); 2 Fondazione Policlinico Universitario Campus Bio-Medico, Via Alvaro del Portillo 200, 00128 Rome, Italy; 3 Unit of Food and Nutrition Science, Department of Science and Technology for Sustainable Development and One Health, Università Campus Bio-Medico di Roma, Via Alvaro del Portillo 21, 00128 Rome, Italy; s.cimini@unicampus.it (S.C.); l.degara@unicampus.it (L.D.G.); 4 The Nano(bio)sensors Lab, Department of Pharmacy, University of Naples Federico II, Via Domenico Montesano 49, 80131 Naples, Italy; stefano.cinti@unina.it

**Keywords:** plant wearables, flexible matrices, plant physiology, photosynthetic efficiency, stomatal conductance

## Abstract

The safeguarding of plant health is vital for optimizing crop growth practices, especially in the face of the biggest challenges of our generation, namely the environmental crisis and the dramatic changes in the climate. Among the many innovative tools developed to address these issues, wearable sensors have recently been proposed for monitoring plant growth and microclimates in a sustainable manner. These systems are composed of flexible matrices with embedded sensing elements, showing promise in revolutionizing plant monitoring without being intrusive. Despite their potential benefits, concerns arise regarding the effects of the long-term coexistence of these devices with the plant surface. Surprisingly, a systematic analysis of their influence on plant physiology is lacking. This study aims to investigate the effect of the color and geometric features of flexible matrices on two key plant physiological functions: photosynthesis and transpiration. Our findings indicate that the negative effects associated with colored substrates, as identified in recent research, can be minimized by holing the matrix surface with a percentage of voids of 15.7%. This approach mitigates interference with light absorption and reduces water loss to a negligible extent, making our work one of the first pioneering efforts in understanding the intricate relationship between plant wearables’ features and plant health.

## 1. Introduction

Safeguarding the health of plants is crucial for maintaining sustainable food production over time. Numerous approaches have been implemented to ensure plant protection, including the proper management of water, pesticides, and fertilizers, the adoption of hydroponic cultivation systems, and the integration of genetically modified plants [1,2,3,4].

Despite the implementation of these agroecosystem-based strategies, the consumption of agrochemicals remains remarkably high, reaching up to 3.5 million tonnes in 2020 [5]. This negatively impacts food quality and environmental security, posing a challenge to the long-term stability and sustainability of agroecosystems.

Over the past few decades, new measuring approaches have emerged to quantitatively assess plant health and reduce yield loss. However, they are often disruptive and characterized by low metrological properties (e.g., low spatial–temporal resolution, sensitivity, stability, and measurement reliability) [6,7].

Therefore, there is an urgent need to develop innovative tools for taking decisive and prompt actions to optimize and scale-up agri-food practices without harming our ecosystem. Against this backdrop, recently, new sensor technologies have been developed to monitor crops by applying innovations in (bio)sensing to plants for improving precision agricultural practices. Just like medical wearables, these new approaches aim to remotely check a plant’s health under a changing environment to provide early signs of stress and optimize crop protection [8]. However, despite the potential of these systems, the development of plant-wearable (bio)sensors is significantly lagging behind, and technological improvements are required.

In the literature, most (bio)sensors for plant health monitoring are wearable systems based on electrical sensors [9,10]. These systems consist of conductive elements enclosed in flexible substrates taped on the plant surface or conductive inks directly brushed on it. Recently, fiber optic sensors have also been proposed for this aim [11,12]. In particular, fiber Bragg gratings (FBGs) have been used to monitor the health of plants [13]. FBGs have been embedded into colored and transparent silicone substrates attached to plant organs (i.e., stems and leaves) [13,14,15]. However, despite their advanced properties and potential, these systems have mainly been used in controlled environments without considering the effect of the interference of the cohabitation of wearable systems and plants on their physiological functions. Two of the main basic physiological processes of plants leaves are photosynthesis, through which plants convert light energy into chemical energy to produce nutrients, and transpiration, which aids in temperature regulation by controlling water loss [16,17,18]. Both processes are essential for sustaining optimal plant health and growth.

Ideally, a wearable system designed for plants should be able to make contact with the plant’s surface without impairing any functions crucial for the plant health. However, alterations in the way in which light is absorbed through the chloroplasts and the water is released through the stomata can occur if external elements are brushed on the plant surface or taped to it.

Considering this crucial aspect, the present study aims to investigate the influence of a constitutive element (i.e., the encapsulation matrix) of wearable plant sensors on photosynthesis and transpiration. The present paper is an extension of the work presented in [19]. In [19], only the influence of the color of the encapsulation matrix was analyzed, and the results showed that, among the various substrates proposed, the transparent one exhibited the least interference with the plant physiological processes. Specifically, photosynthesis remained unaffected, and although a decrease in transpiration was observed, this effect was less pronounced compared to the alterations induced by the colored matrices.

This work expands on our previous one by proposing a crucial change in the substrate geometric features compared with the ones in [19] to mitigate the impact of the matrix color on both photosynthesis and transpiration. We used substrates with the same shape as those in [19], but all the matrices were holed with a percentage of void of 15.7% to allow light to be easily absorbed by the plant and water to transpire from the leaves. This influence was assessed for an acclimation period of 15 days, which can be considered an adequate time to observe a possible effect on photosynthetic efficiency and chlorophyll content in plant leaves due to changes in light exposure [20,21].

## 2. Basics of Plant Photosynthesis and Transpiration

Oxygenic photosynthesis is the process through which solar energy is converted into chemical energy and used to reduce carbon dioxide (CO_2_) into organic compounds. The produced carbohydrates are then exported to satisfy the metabolic needs of the non-photosynthetic cells of the plant, thus ensuring plant growth and development [22,23].

The most active photosynthetic tissue in higher plants is the leaf mesophyll. Different from epidermal cells, mesophyll cells contain chloroplasts, specialized organelles responsible for photosynthesis during the daylight hours. The number of chloroplasts in a mesophyll cell can strongly differ depending on several factors, such as the plant species and environmental conditions like light exposure, in terms of light quality and quantity. The chloroplast number strongly influences the photosynthetic activity of a green tissue and hence plant growth [24,25]. Chlorophylls, pigments located into chloroplasts, are organized into two multi-protein complexes located in the thylakoid membranes [26]. The role of the photosystems is to transform the solar energy into chemical energy usable for all the metabolic processes that life requires. In such a way, photosynthesis renders our planet a thermodynamically open system, able to utilize sun as an endless source of energy. The photosynthetic process consists of several photochemical and enzymatic reactions that take place in chloroplasts, named light and dark reactions. During the first stage, the light phase, the energy derived from sunlight modifies the redox potential of specialized chlorophylls that collect the energy of photons, making the electron able to move along the electron transport chain in the thylakoid membrane. The chlorophyll obtains its electrons from H_2_O producing O_2_ as a secondary product. During this electron transport process, the protons are pumped across the thylakoid membrane, forming an electrochemical proton gradient, which is used by ATP synthase to generate ATP in the stroma. Moreover, high-energy electrons and protons are used to convert NADP+ to NADPH. During the dark phase, also named carbon fixation reactions, NADPH and ATP are used as a reducing power and source of energy, respectively, to drive the conversion of CO_2_ to carbohydrates [22].

The entry of CO_2_ into the mesophyll is a determinant for the activation of the biochemical reactions taking place in the two photosystems. In plants, CO_2_ enters the photosynthetic tissue through stomatal complexes. Stomatal complexes are localized in the epithelium tissue of aerial plant parts and are critical sites for the regulation of gas exchange between the plant and the atmosphere. The stomata also regulate the loss of water vapor from the plant. This process, called transpiration, enables plants to absorb water and mineral nutrients from the soil, allows for their subsequent movement throughout the plant, and improves the plant cooling capacity [27]. The stomata are flanked by two guard cells that control the stomatal aperture size. Guard cells can increase or decrease their turgor status, and this change determines the stomatal opening and closure. The number, size, and distribution of stomata in the epidermis of the different organs influence plant–environment interactions, the aforementioned physiological processes, and plants’ responses to environmental stresses. In fact, the regulation of gas exchange is a determinant for plant survival, since carbon dioxide must be able to penetrate the leaf to allow for photosynthesis, and at the same time, water loss (transpiration) must be minimized to prevent desiccation and wilting/shivering. Plants strongly regulate the turgor status of guard cells, thus controlling the stomatal opening in relation to environmental factors such as temperature, light exposure (light quality and quantity), water availability, and relative humidity. For example, the stomatal opening is induced by low CO_2_ concentrations to facilitate photosynthesis and high humidity. By contrast, closing is promoted by high CO_2_ concentrations, low light exposure, and drought conditions [28,29].

## 3. The Flexible Substrates: Design and Manufacturing

**Figure 1:**
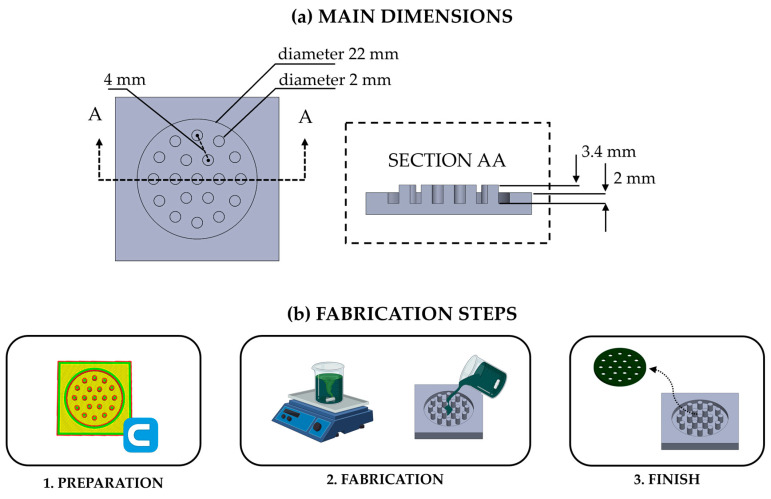
(**a**) The main geometric features and dimensions of the mold for realizing the flexible matrices; (**b**) the main steps of the fabrication process.

The flexible substrates were designed using 3D-printed molds with 19 cylinders concentrically extruded from the mold cavity to create the voids in the matrices. The main dimensions of the designed mold used to shape the flexible substrates were an external diameter of 22 mm, a cylinder diameter of 4 mm, a cylinder extrusion height of 3.4 mm, and a cavity depth of 2 mm; these dimensions were chosen to create 19 holes, corresponding to a percentage of void of 15.7%. For more details, see Figure 1a.

The mold fabrication process can be broken down into three main steps, as shown in Figure 1b:

1. *Preparation*
  A 3D file of the mold is obtained using a 3D design software (Solidoworks 2021) saved in a stl.file. The stl.file is processed using a cutting software (Ultimaker Cura software v5.5.0.) to convert the project into a g.code file that the 3D printer is able to interpret. In the g.code file, the mold is sliced into thin layers and fine-tuned with the chosen printing parameters (i.e., infill density of 60%, printing speed of 40 mm/s, and line patterns).
2. *Fabrication*
  The g.code file is transmitted to the 3D printer (Sovel SV04), and the printing process starts. At the end of the printing process, the mold can be lifted off the printer plate, cleaned, and stripped of the supports. Then, silicone is prepared by mixing Part A and Part B of a silicone rubber (i.e., Dragon skin 20^TM^ commercialized by Smooth-On, Macungie, PA, USA). Dragon skin silicone was chosen since it is one of the most used materials in the fabrication of wearables for both humans and plants [10,30]. Before mixing the two components, pigments (Silc Pig^TM^, Smooth-On, Macungie, PA, USA) are added to Part A of the Dragon skin 20^TM^. The loading percentage is 1% of the total silicone weight. Then, the mixture is degassed to remove the air bubbles trapped in the solution. This process is repeated for all the colored substrates (i.e., pigments: PMS white C, PMS black C, PMS red 186C, PMS green 3292C, and PMS blue 2757C). For the transparent matrix, no pigment is added to Part A.
3. *Finish*
  After 4 h of curing time at ambient temperature, the substrates are peeled out from the molds and ready to be positioned on the plant leaf.

**Figure 2:**
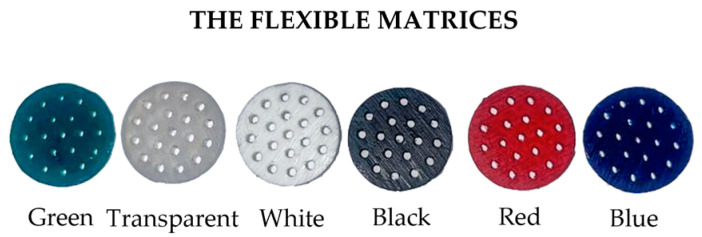
The holed flexible matrices realized in different colors: green, transparent, white, black, red, and blue.

Figure 2 shows all the developed matrices at the end of the fabrication process.

## 4. Analysis of the Influence of Flexible Matrices on Plant Physiology

Two of the main physiological processes of plants are photosynthesis and transpiration [31] (for more information, see Section 2).

### 4.1. Parameters Used to Investigate the Effect on Photosynthesis and Transpiration Efficiency

To analyze the effect of the matrices on photosynthesis and transpiration, this work focuses on the assessment of changes in chlorophyll fluorescence and stomatal conductance.

#### 4.1.1. Chlorophyll a Fluorescence

The measurement of chlorophyll fluorescence is a non-invasive method of assessing photosynthetic efficiency [32] and a commonly used technique in plant physiology. In fact, chloroplasts can absorb light energy and utilize it in three ways: (i) to guarantee the photosynthetic process, (ii) to release it as heat, or (iii) to re-emit it as light in the form of fluorescence [25,33].

The basics of chlorophyll fluorescence analysis are quite straightforward. In essence, the absorption of a photon elevates an electron within chlorophyll molecules to an excited state, and a fluorescence photon is promptly emitted as the molecule returns to its ground state. Nowadays, this measurement can be performed under dark and light conditions [34].

In this study, we used light-adapted measurements to estimate changes in the quantum yield of PSII (Φ_PSII_) [32], calculated as follows:

(1) $$\Phi_{\mathrm{PSII}}={(F}_{m}^{\prime }-F_{s})/F_{m}^{\prime }$$

where $F_{m}^{\prime }$ is the maximum fluorescence in a light-adapted leaf after a saturating pulse of light and $F_{s}$ is the steady-state fluorescence prior to any saturating pulse.

When integrated with stomatal conductance, it enhances the understanding of overall plant physiology and health.

#### 4.1.2. Stomatal Conductance

Stomatal openings play a crucial role in regulating the rate of transpiration (i.e., water loss) and gas exchange (i.e., CO_2_ uptake) through leaves [17,35,36].

In this study, we focus on transpiration; hence, we analyzed the stomatal conductance to water (i.e., *gsw*). *gsw* is a metric influenced by environmental factors such as light, temperature, and humidity and commonly serves as an indicator of the plant water status by analyzing the degree of stomatal opening with respect to the number of stomata.

Stomatal conductance, a measure of the degree of stomatal opening, is generally measured by the use of a porometer [37].

In this case, *gsw* is determined by calculating the apparent transpiration (*E*) using measurements taken within the sampled leaf area in a porometer cuvette and the leaf temperature [38]. In more detail, *E* is derived by measuring the difference between water vapor in an air stream before ($W_{ref}$) and after ${(W}_{sam}$) the porometer interacts with the leaf combined with flow rate (*μ*) and the leaf area (*s*):

(2) $$E=\mu {\;(W}_{sam}-W_{ref})/s$$

The conductance to water vapor (*gtw*) is obtained as a function of *E* and the vapor pressure in the leaf and cuvette. The boundary layer conductance (*gbw*) is calculated as a function of *μ* and cuvette geometry. Finally, *gsw* is obtained as follows:

(3) $$gsw=\frac{1}{\frac{1}{gtw}-\frac{1}{gblw}}$$

### 4.2. Experimental Setup and Protocol

The analysis proposed in this study was carried out as follows. The colored and transparent substrates were placed on the leaf of a sample plant (i.e., *Anthurium andreanum*) grown in a climatic chamber at 24 °C with a photoperiod 16/8 h light/dark. We used the same type of plant as the study in [19] to better compare the obtained results.

**Figure 3:**
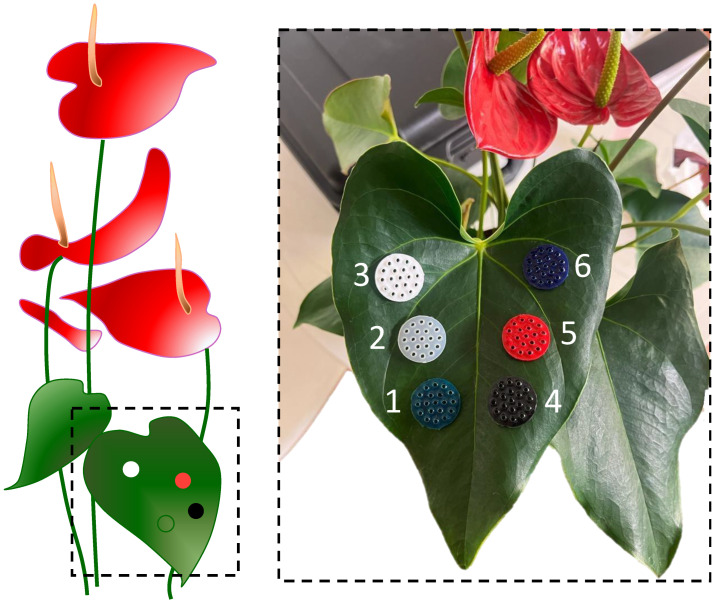
A schematic of the sample plant with a zoomed-in view of the leaf covered by the flexible matrices: green (1), transparent (2), white (3), black (4), red (5), and blue (6).

A soft cloth, dampened with water and squeezed, was delicately wiped on the leaf before positioning the flexible substrates on its surface (see Figure 3).

The effects on photosynthesis and transpiration were evaluated over a period of 15 days using a system (LICOR LI-600 commercialized by Lincoln, NE, USA) integrating a fluorometer to probe Φ_PSII_ and a steady-state porometer for the *gsw* analysis.

Measurements were performed every 2 days at the same time, 2 h after switching on the light. During each measurement, every substrate was removed from the leaf, and the LICOR was used to clamp the leaf area below. Each area was clamped five times. In addition, an area of the leaf uncovered by the developed substrates was also analyzed. From now on, we will refer to this surface as the control area.

### 4.3. Data Analysis and Results

The data recorded by the LICOR instrument were analyzed in a MATLAB R2022a environment to investigate the effects of the proposed substrates on plant physiology.

#### 4.3.1. Chlorophyll a Fluorescence

**Figure 4:**
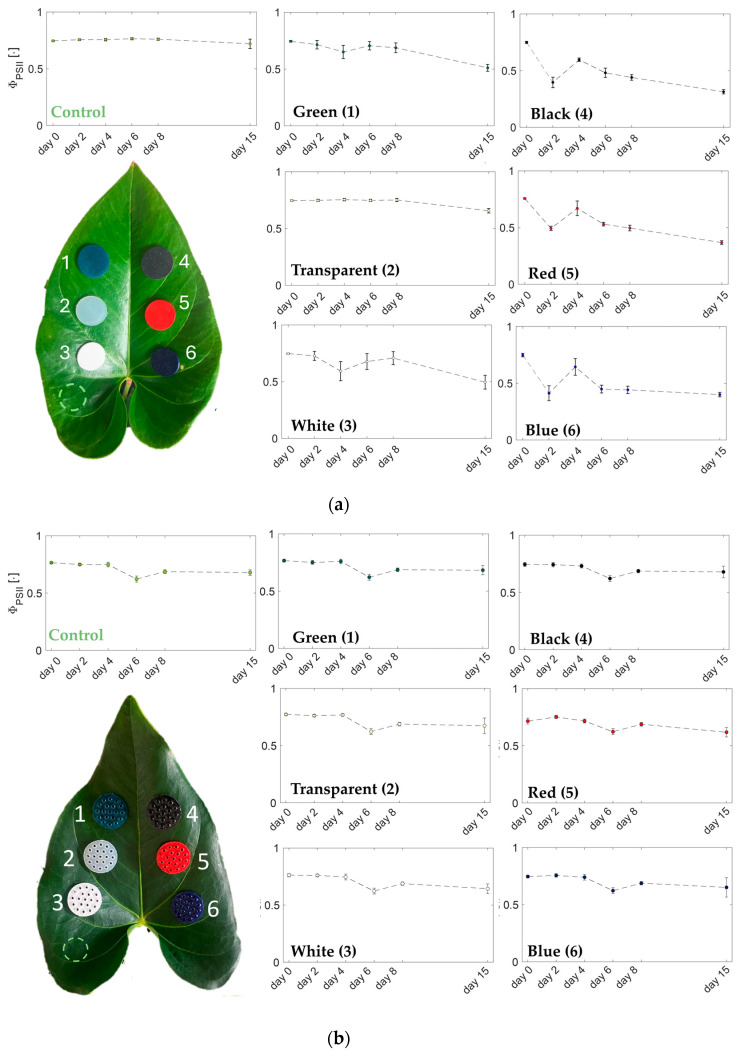
Trends of Φ_PII_ for the flexible substrates positioned on the plant leaf from day 0 to day 8 and after 15 days. The results of the compact ones presented in [19] are shown in (**a**), and those of the holed ones are shown in (**b**).

Regarding the investigation of the effect on photosynthesis, we used the parameter Φ_PSII_, recorded by the LICOR. Figure 4a,b show the trend of Φ_PSII_, expressed as the mean and standard deviation from day 0 to day 8 and after 1 week at day 15 in [19] and in this work, respectively. These values were obtained by averaging the five measurements performed on the control area and the leaf surfaces covered by the flexible matrices. In more detail, each acquisition started with the removal of the substrates from the plant surface. Subsequently, the LICOR was employed to measure Φ_PSII_ in the area where the substrates were positioned, and five consecutive measurements were conducted. Notably, the five measurements for each area were performed on five different points within the designated area.

As clearly visible from the trends in Figure 4b, all the substrates showed values of Φ_PSII_ comparable with the ones probed in the control area. This result emphasized the positive effect of the designed holes in the developed matrices (for more details, see Table 1). In fact, by comparing these findings with the ones in [19], a considerable difference in the trends of Φ_PII_ can be found. The results in [19] showed that the closest value of Φ_PII_ to the one of the control area was found for the transparent matrix, followed by the green and white matrices, while the black, blue, and red substrates showed a different trend (Figure 4a). This suggests that the black, blue, and red pigments have negative influences on the efficiency of photosynthesis. In the present study, as shown in Figure 4b, all the substrates showed a trend of Φ_PII_ similar to the control area, confirming that the presence of the concentric holes in the matrices minimized the effects of the substrates placed on the leaf during photosynthesis [19].

#### 4.3.2. Stomatal Conductance

**Figure 5:**
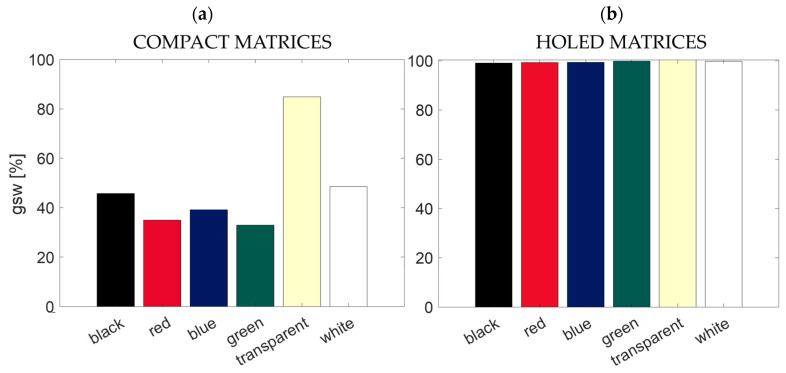
Barplots of the *gsw* values in the area covered by the (**a**) compact [19] and (**b**) holed matrices when positioned on the plant leaf.

The influence of the proposed substrates on the stomatal conductance in terms of *gsw* was investigated as follows. To compare our findings with the ones in [19] (see Figure 5a,b), we conducted a similar analysis. In more detail, all the *gsw* values recorded by the LICOR and related to the areas covered by the colored substrates and the transparent ones were compared with the ones of the control area. Then, we obtained the mean value of *gsw* by averaging all the data related to each matrix to divide by the mean value of *gsw* corresponding to the control area. In this way, the *gsw* value, expressed in percentage, can be obtained as follows:

(4) $$gsw\;[\%]=mean\left(\frac{{gsw}_{flexible\;matrix}}{{gsw}_{control}}\right)\cdot 100$$

The values of gsw obtained in [19] and in the present study are listed in Table 2.

As shown by the results in Figure 5a,b and Table 2, the holes in the substrates also have a positive effect on stomatal conductance. Indeed, by comparing these results with the ones in [19], all the *gsw* values are >99%. Otherwise, in [19], with the exception of the value of *gsw* of the transparent matrix (84.9%), values of 32.9%, 48.5%, 45.7%, 39.1%, and 34.9% were found for the green, white, black, red, and blue substrates, respectively.

## 5. Discussion

This work proposed a change in the design of the flexible substrates to extend the work in [19] by investigating the effect of holed matrix surfaces on both the photosynthetic efficiency and the stomatal conductance of a plant. The results indicate that the placement of the newly designed substrates on the leaf surface does not impact light absorption or water transpiration. Indeed, no appreciable changes in Φ_PII_ were found by analyzing the photosynthetic efficiency changes in both the control area and in the area covered by the developed colored and holed substrates. At day 15, a reduction in terms of mean Φ_PII_ was found for all the analyzed areas. In more detail, reductions of 11.3%, 10.7%, 12.8%, 15.4%, 8.8%, 13.5%, and 12.7% were obtained for mean Φ_PII_ in the control area and those covered by the green, transparent, white, black, red, and blue substrates, respectively. Regarding the changes in gsw, values >99% were always found. In contrast, in [19], the matrices were designed without any holes, and their effects changed depending on the color. The black, blue, and red pigmented substrates negatively affected both the photosynthesis (reductions in mean Φ_PII_ of 58.1%, 51.4%, and 46.6%) and transpiration processes (45.7%, 39.1%, and 34.9%), while the green one predominantly affected stomatal conductance (32.9%), having a smaller impact on photosynthesis (reduction in Φ_PII_ of 31.4%). Notably, the substrate with the weakest effect on both the physiological processes was the transparent one (reduction in Φ_PII_ of 11.9% and gsw > 99%). The results in [19] showed that the effect of wearables on plant physiology changes depending on the color.

In the literature, this aspect has not been systematically investigated. Most of the proposed sensing solutions are based on electrical elements like conductive textiles taped on plants or inks directly brushed on their surface [39,40,41]. For instance, in [39], a wearable sensor consisting of polyacrylic acid double-networked with conductive nanofiller-reduced graphene oxide, coated with polyaniline, and sealed into polydimethylsiloxane was developed to monitor the growth of an *Aloe vera* leaf [39] for 18 days of acquisition. The results showed the potential capacity of the proposed solution to monitor leaf growth over time by detecting the resistance changes. Another example of an electric sensor for plant monitoring was proposed in [40]. This work investigated the use of two strips of carbon nanotube strain gauges placed on the leaf of *Scindapsus aureus* to monitor its growth over 2 days in an indoor environment. The author designed the sensor with a serpentine configuration in mind to optimize the sensitivity to strain and its adaptation to leaf morphology. The first use of a graphene ink for monitoring plant growth was proposed in [41]. In more detail, a graphene ink was directly brushed on two cucumbers (*Cucumis sativus*) to measure changes in electrical resistance for 18 min. The results showed an increase in resistance, along with growth for the sensorized fruits.

Although interesting findings were achieved in the aforementioned studies, most of these studies focused on assessing the working capacity of the proposed sensing solutions without considering the influence of the wearable sensors on plant health. In contrast, these systems are typically black in color and have a compact surface. Consequently, taking into account the analysis carried out in the present study and in [19], it is reasonable to assume that their impact on the basic functions of plants should not be considered negligible. Moreover, this effect is expected to increase with the cohabitation time between the wearable system and the plant surface. For instance, inks are black, and they should not be removed from a plant once brushed on its surface. Hence, a systematic investigation of their effects on plants’ physiological functions should be carried out. The same consideration should be made for conductive elements taped on a plant, especially in the case of long-lasting cohabitations. Indeed, these systems are usually dark in color (e.g., conductive textiles) or metalized (e.g., gold, silver elements). To reduce their effect, considering the results of our study, a certain percentage of void can be realized on their surface. However, holing the surface of these systems can cause unpredictable changes in the electrical signals, consequently inducing measurement unreliability.

To overcome these issues, recently, new systems have been proposed for monitoring plant health. These consist of FBG sensors that are integrated into both colored and transparent matrices before being taped on to a plant’s surface. For instance, in [13], a compact dumbbell-shaped matrix with a light blue color was used to measure the growth of the stem of a *Solanum lycopersicum*. The results were promising, but the tests lasted 12 h in a controlled environment, approximately one day in a real-life scenario. A similar system was proposed in [14], but this system was colored with magenta pigments and placed on the stem of *Nicotiana tabacum* to monitor growth under 2 days of acquisition. In two other studies, a transparent and compact silicone matrix was used to encapsulate FBG sensors and monitor the growth of fruits and leaves. In [42], a ring-shaped matrix was used to measure fruit growth, and in [15], a multi-sensor flower-shaped matrix was used to measure both the growth of a leaf for approximately 4 days and the growth of a fruit for approximately 3 days. Although the results of these works are promising, no investigations of the effect of the proposed flexible wearable sensors based on FBG technology on plant physiology were carried out. However, compared to electrical sensors, FBGs are made of silica and characterized by their high levels of transparency, small size, and light weight. Hence, they could be ideal candidates for the development of plant-wearable sensors with high levels of transparency. Moreover, their miniaturized size (they are extremely thin, like a human hair) makes this technology an ideal candidate for integration into holed transparent matrices. Indeed, FBGs can be easily encapsulated in the portion of the matrix without voids while preserving the integrity of the sensing unit and the substrate itself. This approach will be useful to realize wearable sensors that cause any interferences with the plant physiological processes already investigated in [19] and in the present work.

However, the current study has some shortcomings that need to be addressed in the near future. One issue is our reliance on a single plant species. We anticipated that different plant species might exhibit different times to respond to the application of the matrix in terms of their basic functions. These variations could stem from the differences in the chemical and structural compositions of cell walls, which can vary among taxonomic groups. Therefore, future experiments will investigate these effects across a broader range of plant species. In addition, the effect of other void percentages and size will be analyzed to define the minimum number and size of holes to refine the design of flexible matrices. To delve deeper into this aspect, we will conduct a finite element analysis prior to matrix fabrication. This approach aims to streamline the process by reducing the number of matrices that need to be developed and tested. Finally, FBG sensors will be integrated into the optimized substrate to develop a plant-wearable sensor with a high level of unobtrusiveness.

## 6. Conclusions

This study investigated the effect of plant wearables on plant physiology. We focused on the influence of the flexible matrices that are commonly used to encapsulate different types of sensors (both electrical and optical) on the photosynthesis and transpiration of plants. In particular, this work is an extension of a prior study, [19], in which transparent and colored substrates were developed, and their effect on plant physiology was analyzed. The main novelty of our study lies in its analysis of the influence of transparent and colored substrates with a percentage of void of 15.7%, which was carried out to better investigate whether the presence of holes on the matrix surface can minimize the effect on the basic functions of plant. By comparing the results of the present study with the ones of [19], we can confirm that the negative effect of colored substrates can be reduced by holing their surface. In this way, both the interference with the absorption of light and the loss of water are minimized.

Future tests will be devoted to integrating an FBG sensor into these kinds of substrates and analyzing the effect of the proposed matrix appearance modifications (e.g., percentage of void of 15.7%) on sensor performance. Moreover, an analysis of the influence of different percentages and distributions of voids will be carried out to find the optimal tradeoff between minimal interference with plant health and optimized enclosed sensor performance.

## Figures and Tables

**Table 1:** Values of Φ_PII_ (mean ± standard deviation) from day 0 to day 8 and at 15 days in relation to leaf areas covered by compact [19] and holed matrices.

		Day 0	Day 2	Day 4	Day 6	Day 8	Day 15
Control	COMPACT	0.746 ± 0.007	0.756 ± 0.007	0.756 ± 0.010	0.765 ± 0.009	0.760 ± 0.009	0.720 ± 0.041
	HOLED	0.766 ± 0.048	0.750 ± 0.074	0.749 ± 0.020	0.623 ± 0.026	0.688 ± 0.016	0.679 ± 0.024
Green	COMPACT	0.745 ± 0.007	0.715 ± 0.037	0.651 ± 0.059.	0.706 ± 0.037	0.688 ± 0.043	0.511 ± 0.031
	HOLED	0.766 ± 0.013	0.752 ± 0.016	0.761 ± 0.019	0.623 ± 0.026	0.688 ± 0.016	0.684 ± 0.041
Transparent	COMPACT	0.746 ± 0.007	0.748 ± 0.009	0.755 ± 0.011	0.748 ± 0.009	0.751 ± 0.013	0.657 ± 0.019
	HOLED	0.773 ± 0.008	0.762 ± 0.009	0.768 ± 0.009	0.623 ± 0.026	0.688 ± 0.016	0.674 ± 0.068
White	COMPACT	0.747 ± 0.004	0.728 ± 0.040	0.593 ± 0.084	0.678 ± 0.069	0.709 ± 0.057	0.495 ± 0.061
	HOLED	0.762 ± 0.015	0.759 ± 0.012	0.745 ± 0.025	0.623 ± 0.026	0.688 ± 0.016	0.644 ± 0.041
Black	COMPACT	0.749 ± 0.009	0.397 ± 0.046	0.596 ± 0.015	0.481 ± 0.040	0.440 ± 0.025	0.314 ± 0.019
	HOLED	0.746 ± 0.017	0.744 ± 0.020	0.732 ± 0.018	0.623 ± 0.026	0.688 ± 0.016	0.680 ± 0.051
Red	COMPACT	0.757 ± 0.006	0.492 ± 0.019	0.670 ± 0.064	0.531 ± 0.016	0.496 ± 0.025	0.368 ± 0.015
	HOLED	0.715 ± 0.026	0.752 ± 0.014	0.716 ± 0.017	0.623 ± 0.026	0.688 ± 0.016	0.618 ± 0.041
Blue	COMPACT	0.749 ± 0.015	0.412 ± 0.066	0.645 ± 0.073	0.449 ± 0.034	0.442 ± 0.033	0.400 ± 0.020
	HOLED	0.746 ± 0.012	0.756 ± 0.016	0.738 ± 0.025	0.623 ± 0.026	0.688 ± 0.016	0.651 ± 0.084

**Table 2:** Values of gsw for the compact [19] and holed matrices.

	gsw [%]	
	COMPACT	HOLED
Black	45.7	99.1
Red	39.1	99.3
Blue	34.9	99.3
Green	32.9	99.8
Transparent	99.4	100
White	48.5	99.8

## Data Availability

Dataset available on request from the authors.

## References

[1] Conway G., Toenniessen G. (1999). Feeding the World in the Twenty-First Century. Nature.

[2] United Nations Department of Economics and Social Affairs (2019). World Population Prospects 2019: Highlights|Multimedia Library.

[3] Khreis H. (2020). Traffic, Air Pollution, and Health. Advances in Transportation and Health: Tools, Technologies, Policies, and Developments.

[4] Chartzoulakis K., Bertaki M. (2015). Sustainable Water Management in Agriculture under Climate Change. Agric. Agric. Sci. Procedia.

[5] European Commission Agriculture and the Green Deal. https://commission.europa.eu/strategy-and-policy/priorities-2019-2024/european-green-deal/agriculture-and-green-deal_en.

[6] Kanning M., Kühling I., Trautz D., Jarmer T. (2018). High-Resolution UAV-Based Hyperspectral Imagery for LAI and Chlorophyll Estimations from Wheat for Yield Prediction. Remote Sens..

[7] Mozgeris G., Jonikavičius D., Jovarauskas D., Zinkevičius R., Petkevičius S., Steponavičius D. (2018). Imaging from Manned Ultra-Light and Unmanned Aerial Vehicles for Estimating Properties of Spring Wheat. Precis. Agric..

[8] Qu C.C., Sun X.Y., Sun W.X., Cao L.X., Wang X.Q., He Z.Z. (2021). Flexible Wearables for Plants. Small.

[9] Ang M.C.Y., Lew T.T.S. (2022). Non-Destructive Technologies for Plant Health Diagnosis. Front. Plant Sci..

[10] Lo Presti D., Di Tocco J., Massaroni C., Cimini S., De Gara L., Singh S., Raucci A., Manganiello G., Woo S.L., Schena E. (2023). Current Understanding, Challenges and Perspective on Portable Systems Applied to Plant Monitoring and Precision Agriculture. Biosens. Bioelectron..

[11] Chai Y., Chen C., Luo X., Zhan S., Kim J., Luo J., Wang X., Hu Z., Ying Y., Liu X. (2021). Cohabiting Plant-Wearable Sensor In Situ Monitors Water Transport in Plant. Adv. Sci..

[12] Dufil G., Bernacka-Wojcik I., Armada-Moreira A., Stavrinidou E. (2022). Plant Bioelectronics and Biohybrids: The Growing Contribution of Organic Electronic and Carbon-Based Materials. Chem. Rev..

[13] Lo Presti D., Cimini S., Massaroni C., D’amato R., Caponero M.A., De Gara L., Schena E. (2021). Plant Wearable Sensors Based on FBG Technology for Growth and Microclimate Monitoring. Sensors.

[14] Lo Presti D., Di Tocco J., Massaroni C., Cimini S., Cinti S., D’amato R., Caponero M.A., De Gara L., Schena E. (2022). Fiber Optic Plant Wearable Sensors for Growth and Microclimate Monitoring. Proceedings of the 2022 IEEE International Workshop on Metrology for Industry 4.0 and IoT, MetroInd 4.0 and IoT 2022—Proceedings.

[15] Lo Presti D., Massaroni C., Bianchi D., Di Tocco J., Cimini S., Caponero M.A., Gizzi A., De Gara L., Cinti S., Schena E. (2023). A Wearable Flower-Shaped Sensor Based on Fiber Bragg Grating Technology for In-Vivo Plant Growth Monitoring. IEEE Sens. J..

[16] Tuzet A., Perrier A., Leuning R. (2003). A Coupled Model of Stomatal Conductance, Photosynthesis and Transpiration. Plant Cell Environ..

[17] Damour G., Simonneau T., Cochard H., Urban L. (2010). An Overview of Models of Stomatal Conductance at the Leaf Level. Plant Cell Environ..

[18] De Souza A.P. (2023). Dynamic Responses of Carbon Assimilation and Stomatal Conductance in the Future Climate. J. Exp. Bot..

[19] Lo Presti D., De Tommasi F., Massaroni C., Cimini S., De Gara L., Cinti S., Schena E. (2023). Flexible Wearables for In-Vivo Plant Health Monitoring: The Effect of Colored and Uncolored Substrates on Plant Photosynthesis and Transpiration. Proceedings of the 2023 IEEE International Workshop on Metrology for Industry 4.0 and IoT, MetroInd4.0 and IoT 2023—Proceedings.

[20] Muneer S., Kim E.J., Park J.S., Lee J.H. (2014). Influence of Green, Red and Blue Light Emitting Diodes on Multiprotein Complex Proteins and Photosynthetic Activity under Different Light Intensities in Lettuce Leaves (*Lactuca sativa* L.). Int. J. Mol. Sci..

[21] Liu Z., Mao L., Yang B., Cui Q., Dai Y., Li X., Chen Y., Dai X., Zou X., Ou L. (2023). A Multi-Omics Approach Identifies BHLH71-like as a Positive Regulator of Yellowing Leaf Pepper Mutants Exposed to High-Intensity Light. Hortic. Res..

[22] Amaral J., Lobo A.K.M., Carmo-Silva E. (2024). Regulation of Rubisco Activity in Crops. New Phytol..

[23] Wei Y., Wang S., Yu D. (2023). The Role of Light Quality in Regulating Early Seedling Development. Plants.

[24] Quian-Ulloa R., Stange C. (2021). Carotenoid Biosynthesis and Plastid Development in Plants: The Role of Light. Int. J. Mol. Sci..

[25] Levin G., Schuster G. (2023). LHC-like Proteins: The Guardians of Photosynthesis. Int. J. Mol. Sci..

[26] Lokstein H., Renger G., Götze J.P. (2021). Photosynthetic Light-Harvesting (Antenna) Complexes-Structures and Functions. Molecules.

[27] Bauer H., Ache P., Lautner S., Fromm J., Hartung W., Al-Rasheid K.A.S., Sonnewald S., Sonnewald U., Kneitz S., Lachmann N. (2013). The Stomatal Response to Reduced Relative Humidity Requires Guard Cell-Autonomous ABA Synthesis. Curr. Biol..

[28] Shi Y., Ke X., Yang X., Liu Y., Hou X. (2022). Plants Response to Light Stress. J. Genet. Genom..

[29] Peláez-Vico M.Á., Zandalinas S.I., Devireddy A.R., Sinha R., Mittler R. (2024). Systemic Stomatal Responses in Plants: Coordinating Development, Stress, and Pathogen Defense under a Changing Climate. Plant Cell Environ..

[30] Lo Presti D., Massaroni C., Jorge Leitao C.S., De Fatima Domingues M., Sypabekova M., Barrera D., Floris I., Massari L., Oddo C.M., Sales S. (2020). Fiber Bragg Gratings for Medical Applications and Future Challenges: A Review. IEEE Access.

[31] Farquhar G.D., Sharkey T.D. (1982). Stomatal Conductance and Photosynthesis. Annu. Rev. Plant Physiol..

[32] Earl H.J., Ennahli S. (2004). Estimating Photosynthetic Electron Transport via Chlorophyll Fluorometry without Photosystem II Light Saturation. Photosynth. Res..

[33] Humphrey A.M. (1980). Chlorophyll. Food Chem..

[34] Loriaux S.D., Avenson T.J., Welles J.M., Mcdermitt D.K., Eckles R.D., Riensche B., Genty B. (2013). Closing in on Maximum Yield of Chlorophyll Fluorescence Using a Single Multiphase Flash of Sub-Saturating Intensity. Plant Cell Environ..

[35] Jackson P., Basnayake J., Inman-Bamber G., Lakshmanan P., Natarajan S., Stokes C. (2016). Genetic Variation in Transpiration Efficiency and Relationships between Whole Plant and Leaf Gas Exchange Measurements in *Saccharum* Spp. and Related Germplasm. J. Exp. Bot..

[36] Li S.L., Tan T., Fan Y.F., Raza M.A., Wang Z.L., Wang B., Zhang J.W., Tan X.M., Chen P., Shafiq I. (2022). Responses of Leaf Stomatal and Mesophyll Conductance to Abiotic Stress Factors. J. Integr. Agric..

[37] Ceciliato P.H.O., Zhang J., Liu Q., Shen X., Hu H., Liu C., Schäffner A.R., Schroeder J.I. (2019). Intact Leaf Gas Exchange Provides a Robust Method for Measuring the Kinetics of Stomatal Conductance Responses to Abscisic Acid and Other Small Molecules in Arabidopsis and Grasses. Plant Methods.

[38] von Caemmerer S., Farquhar G.D. (1981). Some Relationships between the Biochemistry of Photosynthesis and the Gas Exchange of Leaves. Planta.

[39] Hsu H.H., Zhang X., Xu K., Wang Y., Wang Q., Luo G., Xing M., Zhong W. (2021). Self-Powered and Plant-Wearable Hydrogel as LED Power Supply and Sensor for Promoting and Monitoring Plant Growth in Smart Farming. Chem. Eng. J..

[40] Zhao Y., Gao S., Zhu J., Li J., Xu H., Xu K., Cheng H., Huang X. (2019). Multifunctional Stretchable Sensors for Continuous Monitoring of Long-Term Leaf Physiology and Microclimate. ACS Omega.

[41] Tang W., Yan T., Ping J., Wu J., Ying Y. (2017). Rapid Fabrication of Flexible and Stretchable Strain Sensor by Chitosan-Based Water Ink for Plants Growth Monitoring. Adv. Mater. Technol..

[42] Lo Presti D., Di Tocco J., Cimini S., Cinti S., Massaroni C., D’Amato R., Caponero M.A., De Gara L., Schena E. (2023). Plant Growth Monitoring: Design, Fabrication, and Feasibility Assessment of Wearable Sensors Based on Fiber Bragg Gratings. Sensors.

